# A Man Who Burst

**Published:** 1878-09

**Authors:** 


A Man who Burst.—We have all of us, in early life, had held up to us as a warning, when too voracious, the terrible story of the boy who ate so much that he burst! The learning of maturer years led us too hastily to discredit this frightful example; for here, in the last number of the *Vierteljahrschrift fur Gerichtliche Medizin*, July, 1878, Dr. Bremuse gives a detailed account of a man who literally burst, split his diaphragm in two, and died, from eating four plates of potato soup, "numerous" cups of tea and milk, followed by a large dose of bicarbonate of soda, to aid digestion. His stomach swelled enormously, and tore the diaphragm on the right side, causing immediate death. The case is probably unique.—*Phil. Med. and Surg. Rep.*

